# Early reports of epidemiological parameters of the COVID-19 pandemic

**DOI:** 10.5365/wpsar.2020.11.3.011

**Published:** 2021-05-11

**Authors:** Keeley Allen, Amy Elizabeth Parry, Kathryn Glass

**Affiliations:** a National Centre for Epidemiology and Population Health, Australian National University, Canberra, Australian Capital Territory, Australia.

## Abstract

**Background:**

The emergence of a new pathogen requires a rapid assessment of its transmissibility, to inform appropriate public health interventions.

**Methods:**

The peer-reviewed literature published between 1 January and 30 April 2020 on COVID-19 in PubMed was searched. Estimates of the incubation period, serial interval and reproduction number for COVID-19 were obtained and compared.

**Results:**

A total of 86 studies met the inclusion criteria. Of these, 33 estimated the mean incubation period (4–7 days) and 15 included estimates of the serial interval (mean 4–8 days; median length 4–5 days). Fifty-two studies estimated the reproduction number. Although reproduction number estimates ranged from 0.3 to 14.8, in 33 studies (63%), they fell between 2 and 3.

**Discussion:**

Studies calculating the incubation period and effective reproduction number were published from the beginning of the pandemic until the end of the study period (30 April 2020); however, most of the studies calculating the serial interval were published in April 2020. The calculated incubation period was similar over the study period and in different settings, whereas estimates of the serial interval and effective reproduction number were setting-specific. Estimates of the serial interval were shorter at the end of the study period as increasing evidence of pre-symptomatic transmission was documented and as jurisdictions enacted outbreak control measures. Estimates of the effective reproduction number varied with the setting and the underlying model assumptions. Early analysis of epidemic parameters provides vital information to inform the outbreak response.

Coronavirus disease 2019 (COVID-19) presents an enormous challenge to public health. By 18 April 2020, 140 million cases had been reported across 222 countries and areas, with an estimate of 3 million people having died. (*1*) The overwhelming attention placed on COVID-19 and the volume of research published in the early months of this pandemic (over 4100 papers in PubMed to the end of April 2020) create challenges for public health responders attempting to understand the epidemiology of this disease. There is a need to distil and synthesize the findings that are most relevant to inform public health interventions.

Estimates of the transmission parameters of a pathogen are required as soon as practicable, to inform the public health response. With known pathogens, public health responders can use data and estimates from previous outbreaks to make evidence-based decisions. However, with an emerging pathogen, such as severe acute respiratory syndrome coronavirus 2 (SARS-CoV-2), past outbreaks may provide limited utility; hence, epidemic parameters must be estimated from early cases and detected transmission events. A successful outbreak response is informed by rapid data collection and analysis, to understand the dynamics of disease spread and identify appropriate, informed interventions.

Understanding disease transmission of a new pathogen requires knowledge of the incubation period, serial interval and reproduction number. The basic reproduction number is the expected or average number of secondary cases that result from one infected person if no individuals in the population are immune to the pathogen and no measures are in place to reduce spread. In practice, pathogens rarely propagate freely through a population because individuals change their behaviour or governments enact public health interventions. The effective reproduction number is the expected or average number of secondary cases in a population where some individuals are immune or interventions to limit spread are in place.

The distribution of the incubation period is crucial for determining the length of quarantine for potentially exposed individuals and travellers. (*2*-*4*) Estimates of the serial interval provide public health responders with an idea of the time available to identify and isolate potential cases before they can spread the disease to others. (*5*, *6*) The reproduction number of a disease provides a population-wide estimate of the scale of a potential outbreak and a baseline to test the effectiveness of different interventions in limiting disease transmission. (*7*-*9*) Although highly influential, early estimates of the incubation period, serial interval and reproduction number are generally based on small sample sizes that may not be representative of the wider population at risk. (*7*, *9*, *10*)

Although some literature reviews have reviewed the epidemiology of COVID-19, (*11*-*14*) they have not collated the estimates of epidemic parameters from the initial period of the COVID-19 pandemic. The aim of this study was to collate and compare the characteristics of the COVID-19 pandemic up to 30 April 2020.

## Methods

Studies that describe or estimate the epidemic characteristics of the COVID-19 pandemic until 30 April 2020 were collected. Epidemiological parameters were limited to the incubation period, the serial interval and the reproduction number. The incubation period is the length of time experienced by an individual case from the point of infection to the start of symptom onset. The serial interval refers to the mean length of time between successive cases in a chain of transmission, measured as the length of time from symptom onset in a primary case to symptom onset in a secondary case. Both the incubation period and serial interval in this analysis are measured in days.

Over the course of the COVID-19 pandemic so far, governments have enacted public health interventions at different times and to different extents. Individual behaviours have changed at different rates as individuals have learned about COVID-19 and responded to media reports, government messaging and their understanding of risk. Several estimates of the reproduction number overlap periods when governments have enacted significant public health interventions. Although this study focuses on estimates from the early stages of the outbreak, when most of the population were susceptible and potentially not modifying their behaviour, this study refers to all estimates of the reproduction number as the effective reproduction number.

We searched peer-reviewed published research articles from PubMed using the terms “coronavirus” AND “novel” OR “new” OR “covid” OR “Wuhan” OR “ncp” OR “ncov” for articles published online until 30 April 2020. The literature search ran from 24 February 2020 to 12 May 2020. All articles were imported to Zotero 5.0.87 for review. Eligible articles were reviewed for date of online publication, study period, sample size, setting, method of calculating epidemic parameters, assumptions used to inform these calculations and output measures (including the approach to estimating uncertainty).

Studies were included in this review if they reported estimates of at least one of the relevant epidemic parameters and were written in English. Any articles published before 1 November 2019, pre-prints, grey literature and case reports were excluded.

### Ethics and permissions

Ethical approval was not sought for this review of existing, publicly available peer-reviewed literature.

## Results

**Figure 1:**
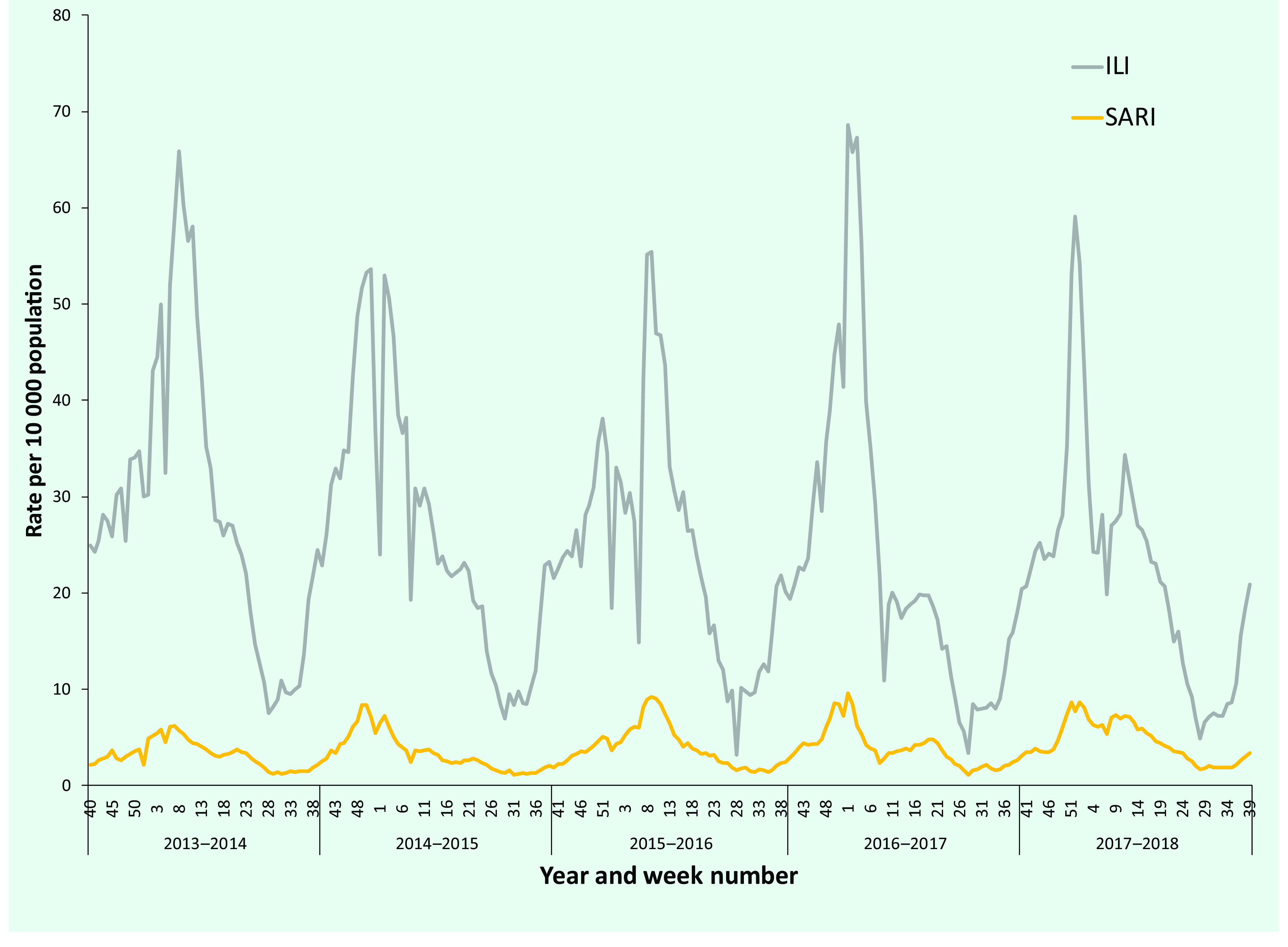
Preferred reporting items for systematic reviews and meta-analysis diagram of study selection

The PubMed search returned 4426 articles published online up to 30 April 2020. Of these articles, 3581 were excluded at the screening assessment and a further 759 at the eligibility assessment, giving a total of 86 included studies. The results of the search and eligibility assessment are shown in **Fig. 1**.

[**insert** Figure 1]

Of the 86 included studies, 15 calculated more than one epidemic parameter of interest. Sixty of the 86 studies used data from mainland China for part or all of their analysis, and 11 specifically analysed outbreak data from Hubei province or the city of Wuhan.

### Incubation period

A total of 33 studies estimated the incubation period of COVID-19 (**Table 1**). Mean estimates were reported in 15 studies, ranging from 1.8 to 9.9 days; however, 44% of the mean estimates were 5–6 days. The shortest mean estimate (incubation period = 1.8 days) was calculated from returned travellers from Hubei province in China, using their last day of travel as their date of exposure. (*29*) One study’s mean estimate of 9.9 days was calculated from a series of 14 cases in Viet Nam. (*33*)

**Table 1:** Estimated incubation period of COVID-19 from included epidemiological parameters studies published between 1 January and 30 April 2020

Study authors	Online publication date	Study period	Sample size	Setting	Estimate (days)*	Uncertainty estimate (days)	Uncertainty measure
Chan et al. (*15*)	24 January 2020	26 December 2019 –15 January 2020	5	Mainland China	-	3–6	Range
Li et al. (*16*)	29 January 2020	Up to 22 January 2020	10	Wuhan/Hubei	5.2	4.1–7.0	95% CI
Backer, Klinkenberg and Wallinga (*17*)	6 February 2020	20 January 2020 –28 January 2020	88	International	6.4	5.6–7.7	95% CrI
K_i_ and Task Force for 2019-nCoV (*18*)	9 February 2020	20 January 2020 –8 February 2020	28	Republic of Korea	3.9; [3.0]	0–15	Range
Jiang, Rayner and Luo (*19*)	13 February 2020	Up to 8 February 2020	50	Mainland China	4.9	4.4–5.5	95% CI
Linton et al. (*20*)	17 February 2020	17 December 2019 –31 January 2020	158	International	5.6; [4.6]	4.4–7.4; 3.7–5.7	95% CrI
Xu et al. (*21*)	19 February 2020	10 January 2020 –26 January 2020	56	Mainland China	[4]	3–5	IQR
Tian et al. (*22*)	27 February 2020	20 January 2020 –10 February 2020	203	Mainland China	[6.7]	± 5.2	SD
Cai et al. (*23*)	28 February 2020	19 January 2020 –3 February 2020	10	Mainland China	6.5	2–10	Range
Guan et al. (*24*)	28 February 2020	Up to 23 January 2020	291	Mainland China	[4]	2–7	IQR
Liu et al. (*25*)	3 March 2020	1 January 2020 –5 February 2020	58	Mainland China	6.0; [5.0]	3–8; 1–16	IQR; Range
Lauer et al. (*26*)	10 March 2020	4 January 2020 –24 February 2020	181	International	[5.1]	4.5–5.8	95% CI
Zhao et al. (*27*)	12 March 2020	23 January 2020 –5 February 2020	19	Mainland China	[8]	6–11	IQR
Pung et al. (*28*)	16 March 2020	18 January 2020 –10 February 2020	17	Singapore	[4]	3–6; 1–11	IQR; Range
Leung (*29*)	18 March 2020	20 January 2020 –12 February 2020	105	Mainland China (travelled to Hubei)	1.8	1.0–2.7	95% CI
			70	Mainland China (local transmission)	7.2	6.1–8.4	95% CI
Chang et al. (*30*)	23 March 2020	28 January 2020 –9 February 2020	15	Mainland China	[5]	1–6	Range
Jin et al. (*31*)	24 March 2020	17 January 2020 –8 February 2020	21	Mainland China – GI symptoms	[4]	3–7	IQR
			195	Mainland China – No GI symptoms	[5]	3–8	IQR
Zhang et al. (*32*)	2 April 2020	19 January 2020 –17 February 2020	49	Mainland China	5.2	1.8–12.4	95% CI
Le et al. (*33*)	2 April 2020	17 January 2020 –14 February 2020	12	Viet Nam	9.9	± 5.2	SD
Zhu and Chen (*34*)	2 April 2020	1 December 2019 –23 January 2020	Not specified	Mainland China, Hong Kong Special Administrative Region (SAR) China, Macau (SAR) China, Taiwan (China)	5.67	1–14	Range
Han et al.35	6 April 2020	31 January 2020 –16 February 2020	25	Mainland China – adults	[5]	3–12	Range
			7	Mainland China – children	[4]	2–12	Range
Shen et al.36	7 April 2020	8 January 2020 –26 February 2020	6	Mainland China	[7.5]	1–16	Range
Sanche et al.37	7 April 2020	15 January 2020 –30 January 2020	24	Mainland China	4.2	3.5–5.1	95% CI
Ghinai et al.38	8 April 2020	February–March 2020	15	United States of America	4.3; [4]	1–7	Range
Huang et al.39	10 April 2020	23 January 2020 –20 February 2020	8	Mainland China	[2]	1–4	Range
Zheng et al.40	10 April 2020	17 January 2020 –7 February 2020	161	Mainland China	[6]	3–8	Range
Xia et al.41	12 April 2020	23 January 2020 –18 February 2020	10	China incl. Hong Kong Special Administrative Region (SAR) China, Macau (SAR) China, Taiwan (China)	7.0	± 2.59; 2–14	SD; Range
Chen et al.42	14 April 2020	28 January 2020 –11 February 2020	12	Mainland China	8.0	1–13	Range
Song et al.43	23 April 2020	16 January 2020 –29 January 2020	22	Mainland China	-	2–13	Range
Jiang et al.44	23 April 2020	23 January 2020 –13 February 2020	4	Mainland China	-	9–13	Range
Nie et al.45	27 April 2020	19 January 2020 –8 February 2020	2907	Mainland China	[5]	2–8	IQR
Yu et al.46	29 April 2020	Up to 19 February 2020	132	Mainland China	[7.2]	6.4–7.9	95% CI
Bi et al.47	30 April 2020	14 January 2020 –12 February 2020	138	Mainland China	[4.8]	4.2–5.4	95% CI

*Mean estimates. Median estimates are shown in [square brackets]. Multiple estimates of incubation period for the same population within the same study are shown in the same row and separated by a semicolon. Estimates of the incubation period in the same study for different populations are shown in separate rows.

CI: confidence interval; CrI: credible interval; GI: gastrointestinal; IQR: interquartile range; SD: standard deviation.

Notes: Sample size reported in Table 1 is the sample size used to calculate the incubation period, not necessarily the whole study sample. All estimates are reported to one decimal place, except where stating findings from papers that did not provide that level of precision.

**Figure 2:**
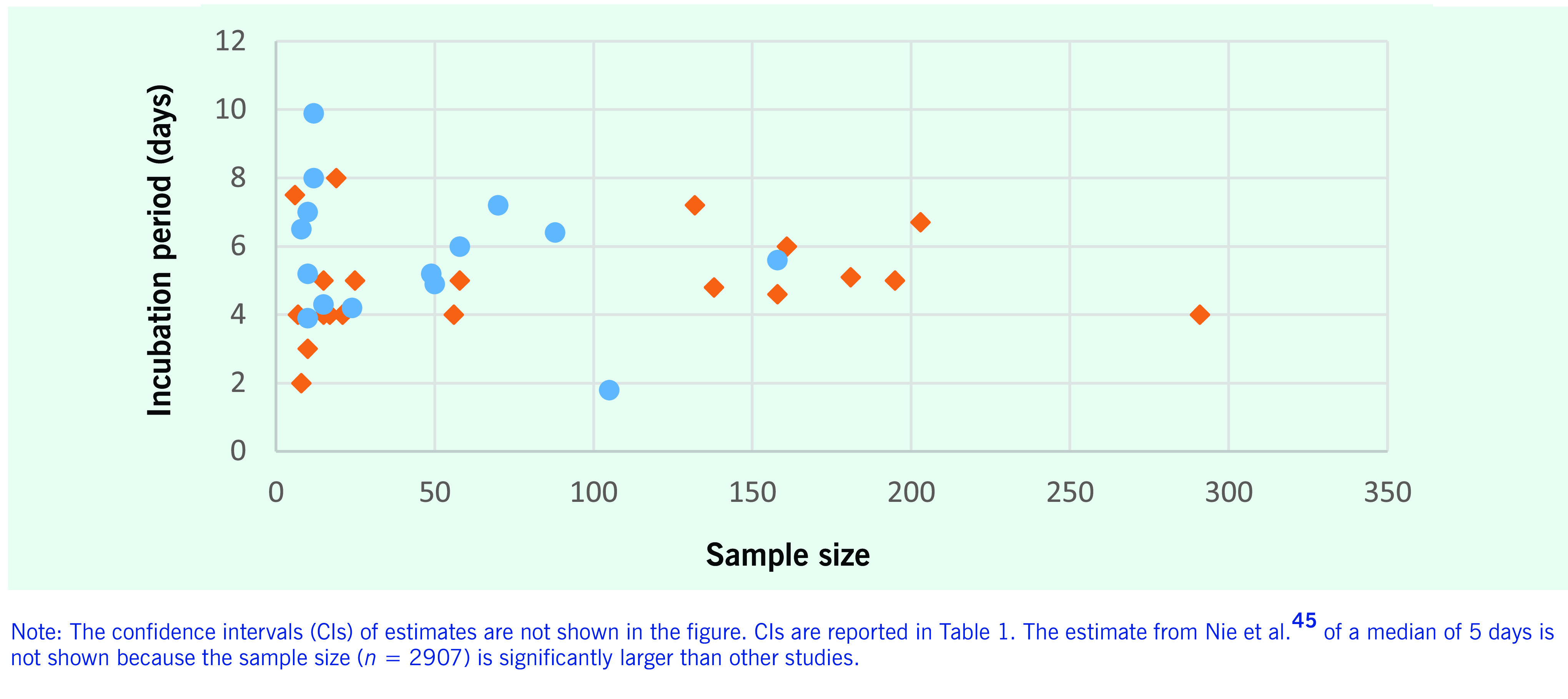
Incubation period estimates and sample size of study (n = 28 studies, 35 estimates) published between 1 January and 30 April 2020

A further 22 estimates of the incubation period were summarized by their median. These studies were generally reporting on a specific cluster or outbreak investigation, and median estimates largely ranged from 4 to 7 days. Estimates outside of this range were calculated from case series; for example, a median range of 1–4 days was found among eight participants (*35*) and an estimated 8-day incubation period for a study involving 19 participants. (*27*) The distribution of the mean and median incubation estimates by sample size of the study is shown in **Fig. 2**.

[**insert** Figure 2]

A further three studies only included a range of observed incubation periods. The longest incubation period from these studies was 16 days, recorded in an outbreak investigation in mainland China. (*36*) Additional estimates of the 95th percentile of the incubation period ranged from 10.3 days (95% confidence interval [CI]: 8.6–14.1) (*17*) to 14 days (95% CI: 12.2–15.9). (*37*)

### Serial interval

Of the 15 studies that included a serial interval, eight were published in April 2020. Mean serial interval estimates were calculated in 14 studies and ranged from 3.1 to 7.5 days (**Table 2**).

**Table 2:** Estimated serial interval from included COVID-19 epidemiological parameters studies published between  1 January and 30 April 2020

Study authors	Online publication date	Study period	Sample size	Transmission pairs	Setting	Estimate (days)*	Uncertainty estimate (days)	Uncertainty measure
Li et al. (*16*)	29 January 2020	Up to 22 January 2020	10	6	Wuhan/Hubei	7.5	5.3–19.0	95% CI
K_i_ and Task Force for 2019-nCoV (*18*)	9 February 2020	20 January 2020 –8 February 2020	28	12	Republic of Korea	6.6; [4.0]	3–15	Range
Liu et al. (*25*)	3 March 2020	1 January 2020 –5 February 2020	15 single intracluster transmission cases	12 clusters	Mainland China	5.5	-	-
			56 single co-exposure cases	56 clusters	Mainland China	3.1	-	-
Nishiura et al. (*38*)	4 March 2020	Up to 12 February 2020	Not specified	28 – all pairs	International	[4.0]	3.1–4.9	95% CrI
				18 – most certain pairs	International	[4.6]	3.5–5.9	95% CrI
Pung et al. (*28*)	16 March 2020	Up to 15 February 2020	4	3	Singapore		3–8	Range
Du et al. (*39*)	19 March 2020	21 January 2020 –8 February 2020	752	468	Mainland China	4.0	3.5–4.4	95% CI
Wu et al. (*40*)	19 March 2020	1 December 2019 –28 February 2020	Not specified	43	International	7	5.8–8.1	95% CI
Zhang et al. (*32*)	2 April 2020	19 January 2020 –17 February 2020	63	35	Mainland China	5.1	3.1–11.6	95% CI
Ji et al. (*41*)	7 April 2020	23 January 2020 –27 March 2020	51	32	Wuhan/Hubei	6.5	6.3	SD
Huang et al. (*35*)	10 April 2020	23 January 2020 –20 February 2020	9	8	Mainland China	[1]	0–4	Range
Wang et al. (*42*)	10 April 2020	11 January 2020 –16 February 2020	115	85	Wuhan/Hubei	5.5	± 2.7	SD
He et al. (*43*)	15 April 2020	7 January 2020 –4 March 2020	Not specified	77	International	5.8; [5.2]	4.8–6.8; 4.1–6.4	95% CI
Kwok et al. (*44*)	23 April 2020	23 January 2020 –13 February 2020	38	26	Hong Kong Special Administrative Region (SAR) China	4.6	3.4–5.9	95% bCI
				26 – adjusted for right truncation	Hong Kong Special Administrative Region (SAR) China	4.8	3.5–6.9	95% CrI
Bi et al. (*37*)	27 April 2020	14 January 2020 –12 February 2020	Not specified	48	Mainland China	6.3; [5.4]	5.2–7.6; 4.4–6.5	95% CI
Ganyani et al. (*45*)	30 April 2020	14 January 2020 –27 February 2020	54	4 clusters	Singapore	5.2	–3.4–13.9	95% CrI
			114	16 clusters	Mainland China	3.9	–4.5–12.5	95% CrI

*Mean estimates. Median estimates are shown in [square brackets]. Multiple estimates of serial interval for the same population within the same study are shown in the same row and separated by a semicolon. Estimates of the serial interval in the same study for different populations are shown in separate rows.

bCI: Bayesian confidence interval; CI: confidence interval; CrI: credible interval; SD: standard deviation.

Notes: Sample size reported is the sample size used to calculate the serial interval, not necessarily the whole study sample. All estimates are reported to one decimal place, except where stating findings from papers that did not provide that level of precision.

The estimated serial intervals were longer in studies published at the start than at the end of the study period, with a mean interval of 7.5 days in late January 2020 and a mean of 4–5 days in early March 2020. Estimates published from March 2020 onwards included transmission pairs with negative serial intervals, or intervals shorter than the incubation period, suggesting possible pre-symptomatic transmission. Mean estimates of the serial interval that included negative transmission pairs generally ranged from 3.9 to 5.8 days (**Table 2**).

The four median serial interval estimates ranged from 1.0 to 5.4 days. Excluding the estimate of 2 days from a case series of eight cases, (*35*) the median serial interval ranged from 4.0 to 5.4 days (**Table 2**).

### Reproduction number

There were 90 estimates of the reproduction number from 52 studies across three World Health Organization (WHO) regions: Western Pacific Region, European Region and Region of the Americas. Reproduction number estimates ranged from 0.3 to 14.8. Of the 90 reported estimates, 33 estimates (37%) were between 2 and 3, and 20 estimates (22%) were between 3 and 4 (**Table 3**).

**Table 3:** Estimated reproduction number from included COVID-19 epidemiological parameters studies published between 1 January and 30 April 2020

Study authors	Online publication date	Study period	Sample size	Method	Setting	Estimate	Uncertainty interval	Uncertainty measure
Wu et al. (*46*)	23 January 2020	10 January 2020 –12 January 2020	41	Zoonotic transmission – Cauchemez et al. 2013 (*47*)	Wuhan/Hubei	0.3	0.17–0.44	95% CI
Li et al. (*16*)	29 January 2020	Up to 22 January 2020	425	Transmission model with renewal equations	Wuhan/Hubei	2.2	1.4–3.9	95% CI
Riou and Althaus (*48*)	30 January 2020	Up to 18 January 2020	50	Stochastic transmission model	Wuhan/Hubei	2.2	1.4–3.8	90% HDI
Zhao et al. (*49*)	30 January 2020	10 January 2020 –24 January 2020	2033	Exponential growth model method	Mainland China	2.24 –3.58	1.96–2.55 to 2.89–4.39	95% CI
Wu et al. (*50*)	31 January 2020	1 December 2019 –28 January 2020	55	Differential equation – SEIR compartment model	International	2.68	2.47–2.86	95% CrI
Zhao et al. (*51*)	1 February 2020	1 December 2019 –24 January 2020	41	Exponential growth model method	Mainland China	2.56	2.49–2.63	95% CI
Tang et al. (*52*)	7 February 2020	10 January 2020 –15 January 2020	41	Differential equation – SEIR compartment model	Mainland China	6.47	5.71–7.23	95% CI
K_i_ and Task Force for 2019-nCoV (*18*)	9 February 2020	20 January 2020 – 8 February 2020	26	Estimated from transmission chains	Republic of Korea	0.48	0.25–0.84	95% CI
Zhou et al. (*53*)	12 February 2020	Up to 25 January 2020	2820	Differential equation – SEIR compartment model	Mainland China	2.83–3.28	-	-
Jung et al. (*54*)	14 February 2020	31 December 2019 –24 January 2020	92	Exponential growth model method	Mainland China	2.1; 3.2	2.0–2.2; 2.7–3.7	95% CI
Zhang et al. (*55*)	22 February 2020	Up to 16 February 2020	355	Cori et al. methodology (*56*)	Cruise ship	2.28	2.06–2.52	95% CI
Lai et al. (*57*)	25 February 2020	Up to 4 February 2020	52	Coalescent-based exponential growth and a birth-death skyline method	Mainland China	2.6	2.1–5.1	95% CI
Chen et al. (*58*)	28 February 2020	7 December 2019 –1 January 2020	Not specified	Bats-Hosts-Reservoir-People transmission network model	Wuhan/Hubei	3.58	-	-
Rocklov, Sjodin and Wilder-Smith (*59*)	28 February 2020	21 January 2020 –19 February 2020	3700	Differential equation – SEIR compartment model	Cruise ship	14.8	-	-
Mizumoto and Chowell (*60*)	29 February 2020	20 January 2020 –17 February 2020	3711	Discrete time integral equation	Cruise ship	5.8	0.6–11.0	95% CrI
Fang, Nie and Penny (*61*)	6 March 2020	20 January 2020 –29 February 2020	35 329	Differential equation – SEIR compartment model	Mainland China	2.35–3.21	-	-
Zhou et al.70	10 March 2020	10 January 2020 –31 January 2020	44	Differential equation – SEIR compartment model	Mainland China	5.3167	-	-
Kucharski et al.71	11 March 2020	1 December 2019 –11 February 2020	Not specified	Differential equation – SEIR compartment model	Wuhan/Hubei	2.35	1.15–4.77	95% CI
Yang and Wang72	11 March 2020	23 January 2020 –10 February 2020	Not specified	Differential equation – SEIR compartment model	Wuhan/Hubei	4.25	-	-
Zhao and Chen73	11 March 2020	20 January 2020 –30 January 2020	Not specified	Differential equation – SEIR compartment model	Mainland China	4.7092	-	-
Choi and Ki74	12 March 2020	29 December 2019 –3 January 2020	Not specified	Differential equation – SEIR compartment model	Wuhan/Hubei	4.028	4.010–4.046	95% CI
-	-	20 January 2020 –17 February 2020	30	-	Republic of Korea	0.555	0.509–0.602	95% CI
Kuniya75	13 March 2020	15 January 2020 –29 February 2020	239	Differential equation – SEIR compartment model	Japan	2.6	2.4–2.8	95% CI
Remuzzi and Remuzzi76	13 March 2020	19 February 2020 –8 March 2020	Unclear	Exponential growth model method	Italy	2.76–3.25	-	-
Li et al.77	16 March 2020	10 January 2020 –23 January 2020	801	Differential equation – SEIR compartment model	Mainland China	2.38	2.03–2.77	95% CrI
Shim et al.78	17 March 2020	20 January 2020 –26 February 2020	6284	Generalized growth model	Republic of Korea	1.5	1.4–1.6	95% CI
Du et al.49	19 March 2020	21 January 2020 –8 February 2020	752	Not stated	Mainland China	1.32	1.16–1.48	95% CI
Wu et al.50	19 March 2020	1 December 2019 –28 February 2020	45 771	Differential equation – SEIR compartment model	Wuhan/Hubei	1.94	1.83–2.06	95% CrI
Yuan et al.79	28 March 2020	23 February 2020 –9 March 2020	Not specified	Exponential growth model method; Wallinga time dependent method	Italy	3.27; 3.10	3.17–3.38; 2.21–4.11	95% CI
-	-	-	-	-	France	6.32; 6.56	5.72–6.99; 2.04–12.26	95% CI
-	-	-	-	-	Spain	5.08; 3.95	4.51–5.74; 0–10.19	95% CI
-	-	-	-	-	Germany	6.07; 4.43	5.51–6.69; 1.83–7.92	95% CI
Anastassopoulou et al.80	31 March 2020	11 January 2020 –10 February 2020	Not specified	Differential equation – SEIR compartment model	Wuhan/Hubei	4.6	3.56–5.65	90% CI
Ferretti et al.81	31 March 2020	Up to end March 2020	40 transmission pairs	Exponential growth model method	Mainland China	2	1.7–2.5	90% CI
Huang et al.82	31 March 2020	13 January 2020 –9 March 2020	80 754	Differential equation – SEIR compartment model	Mainland China	2.23–2.51	-	-
Tian et al.83	31 March 2020	31 December 2019 –23 January 2020	Not specified	Differential equation – SEIR compartment model	Mainland China	3.15	3.04–3.26	95% BCI
Zhu and Chen34	2 April 2020	1 December 2019 –23 January 2020	Not specified	Poisson Transmission Model	Mainland China	2.47	2.39–2.55	95% CI
Sanche et al.37	7 April 2020	15 January 2020 –30 January 2020	140	Differential equation – SEIR compartment model	Mainland China	5.7	3.8–8.9	95% CI
Zhao et al.84	8 April 2020	1 December 2019 –8 January 2020	Not specified	Differential equation – SEIR compartment model	Wuhan/Hubei	2.5	2.4–2.7	95% CI
Pan, Liu and Wang85	10 April 2020	5 December 2019 –8 March 2020	32 583	Cori et al. methodology112	Wuhan/Hubei	3.82	3.72–3.93	95% CrI
Abbott et al.86	14 April 2020	Up to 25 January 2020	1975	Stochastic branching process model	Mainland China	2.8–3.8	-	-
Puci et al.	14 April 2020	22 March 2020 –29 March 2020	975	Differential equation – SEIR compartment model	Italy	1.82	1.51–2.01	95% CI
Du et al.87	16 April 2020	1 December 2019 –22 January 2020	19	Exponential growth method	Mainland China	1.9	1.47–2.59	95% CrI
Torres-Roman et al.88	17 April 2020	6 March 2020 –15 March 2020	Not specified	Cori et al. methodology112	Peru	2.97	-	-
Tsang et al.89	20 April 2020	15 January 2020 –3 March 2020	Not specified	Exponential growth model	Mainland China	2.8–3.5	-	-
Muniz- Rodriguez et al.90	22 April 2020	19 February 2020 –19 March 2020	978	Exponential growth model; renewal equations method	Islamic Republic of Iran	4.4; 3.5	3.9–4.9; 1.3–8.1	95% CI
Zhuang et al.91	22 April 2020	Up to 5 March 2020	Not specified	Stochastic model, maximum likelihood estimation approach	Italy	2.6; 3.3	2.3–2.9; 3.0–3.6	95% CI
-	-	-	-	-	Republic of Korea	2.6; 3.2	2.3–2.9; 2.9–3.5	95% CI
Gatto et al.92	23 April 2020	24 February 2020 –23 March 2020	107	Differential equation – SEIR compartment model	Italy	3.6	3.49–3.84	95% CI
Han et al.93	23 April 2020	21 January 2020 –15 February 2020	482	Exponential growth model method	Mainland China	2.9	1.8–4.5	95% CI
Caicedo-Ochoa et al.94	25 April 2020	Up to 23 March 2020 (first 10 days after reaching 25 cases in each location)	Not specified	Cori et al. methodology112 Two serial intervals used: 7.5 days; 4.7 days	Spain	6.48; 2.9	5.97–7.02; 2.67–3.14	95% CrI
-	-	-	-	-	Italy	6.41; 2.83	6.11–6.71; 2.70–2.96	95% CrI
-	-	-	-	-	Ecuador	12.86; 3.95	12.05–13.68; 3.70–4.21	95% CrI
-	-	-	-	-	Panama	7.19; 3.67	6.37–8.08; 3.25–4.13	95% CrI
-	-	-	-	-	Brazil	6.53; 2.91	5.85–7.25; 2.60–3.23	95% CrI
-	-	-	-	-	Chile	5.79; 2.67	5.32–6.28; 2.45–2.89	95% CrI
-	-	-	-	-	Colombia	5.65; 2.67	5.04–6.29; 2.38–2.98	95% CrI
-	-	-	-	-	Peru	5.24; 2.36	4.68–5.83; 2.11–2.63	95% CrI
-	-	-	-	-	Mexico	4.94; 2.42	4.37–5.56; 2.14–2.72	95% CrI
Bi et al.47	27 April 2020	14 January 2020 –12 February 2020	48	Estimated from transmission chains	Mainland China	0.4	0.3–0.5	95% CI
Distante et al.95	27 April 2020	Up to 29 March 2020	Not specified	Exponential growth method	Italy	3.6	-	-
Ndairou et al.96	27 April 2020	4 January 2020 –9 March 2020	Not specified	Differential equation – SEIR compartment model	Wuhan/Hubei	0.945	-	-
Peirlinck et al.97	27 April 2020	21 January 2020 –4 April 2020	311 357	Differential equation – SEIR compartment model	United States of America	5.3	± 0.95	SD
Adegboye et al.98	28 April 2020	27 February 2020 –11 April 2020	318	Cori et al. methodology112	Nigeria	2.71	-	-
Ganyani et al.55	30 April 2020	14 January 2020 –27 February 2020	91	Exponential growth model method	Singapore	1.25	1.17–1.34	95% CrI
-	-	-	135	Exponential growth model method	Mainland China	1.41	1.26–1.58	95% CrI
Ivorra et al.99	30 April 2020	1 December 2019 –29 March 2020	Not specified	Differential equation – SEIR compartment model	Mainland China	4.2732	-	-

Multiple estimates of the reproduction number for the same population within the same study are shown in the same row and separated by a semicolon. Estimates of the incubation period in the same study for different populations are shown in separate rows.

bCI: Bayesian confidence interval; CI: confidence interval; CrI: credible interval; HDI: high density interval; SD: standard deviation; SEIR: susceptible-exposed-infected-recovered.

Notes: Sample size reported is the sample size used to calculate the serial interval, not necessarily the whole study sample. All estimates are reported to the number of decimal places provided in each study.

The initial low estimate of 0.3 relied on the early assumption that the pathogen was primarily spread through zoonotic transmission. (*46*) Other estimates of the reproduction number under 1 were reported in jurisdictions with rapid public health interventions during the study period, including the Republic of Korea and Singapore. (*18*, *45*, *62*) The highest reproduction number estimate (14.8) was from analyses of transmission dynamics onboard the Diamond Princess cruise ship. (*59*)

**Figure 3:**
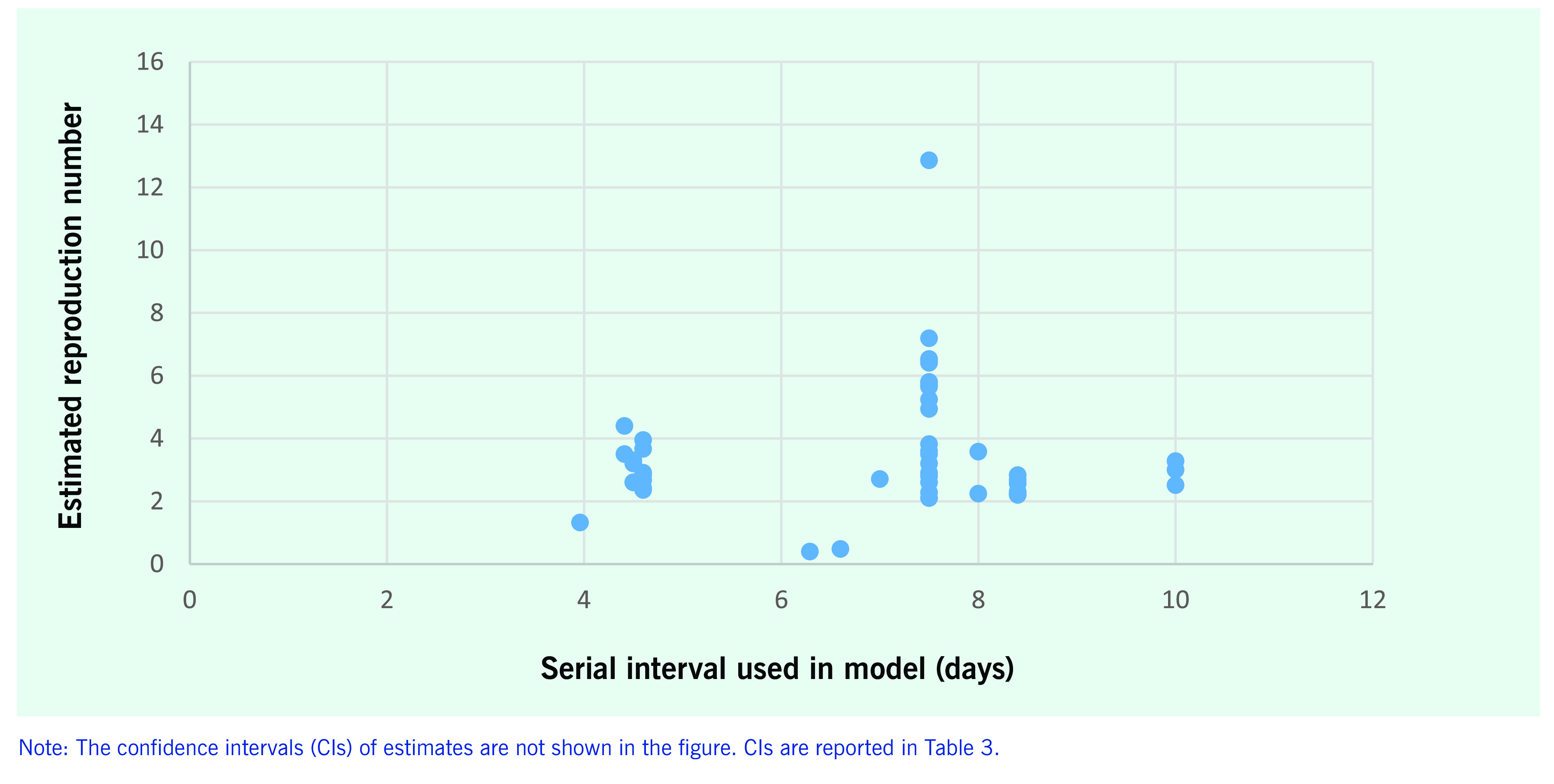
Estimated reproduction number and serial interval of the model (n = 23 studies, 50 estimates) published between 1 January and 30 April 2020

The distribution of reproduction number estimates by the assumed serial interval is shown in **Fig. 3**. Just over half (*n* = 50) of the 90 reproduction number results used an estimate of the serial interval to calculate the reproduction number. Serial interval estimates used to estimate the reproduction number ranged from 4 (*39*) to 10 days, with the latter taken from the estimated serial interval for severe acute respiratory syndrome (SARS) in early outbreaks. (*63*) Studies generally applied serial intervals from the earliest COVID-19 estimate of 7.5 days (*16*) and the accepted serial interval of SARS of 8.4 days. (*63*)

[**insert** Figure 3]

## Discussion

This study provides a review of estimated epidemic parameters of the COVID-19 outbreak up to 30 April 2020. Estimates of the incubation period were similar across the study period, with a mean estimated value of 5–6 days and a range of 2–14 days. Estimates of the serial interval shortened over the study period, from 7.5 days in late January 2020 to a mean of 4–5 days in early March 2020.

Estimates of the reproduction number varied in the studies collated up to 30 April 2020. Although some estimates of the reproduction number were as high as 14.8, over half were between 2 and 4. The higher estimates demonstrate the impact of the setting, individual behaviours and public health interventions – the highest estimates were associated with cruise ships, (*55*, *59*, *60*) whereas the lowest estimates were generally calculated in areas with a rapid response to an outbreak. (*18*, *45*, *62*, *64*)

The incubation period reflects the growth of a virus in an individual, and thus is largely a biological function that would not be expected to vary with changes in human behaviour and wider public health interventions. Variations in the incubation period reported in this study may, in part, result from the study designs adopted. Several estimates of the incubation period were reported directly from cluster investigations, often with low sample sizes. Studies with more than 20 participants had less variation between estimates than studies with smaller sample sizes. The definition of exposure, including the potential for continuous exposure in a household, may also have influenced results by artificially lengthening or shortening the incubation period, depending on study design and differences in local epidemiological reporting protocols.

The serial interval and reproduction number are likely to be influenced by public health interventions, social behaviours and political decisions. Estimates of these two epidemic characteristics are therefore setting-specific, which may explain the variance across the results in this study. The serial interval estimates also changed as new information about the pathogen came to light, primarily the potential for pre-symptomatic and pauci-symptomatic transmission. (*65*-*70*) However, these revised estimates of the serial interval were rarely used to revise reproduction number estimates. A longer serial interval results in a higher estimate of the reproduction number. The earliest published estimate by Li et al.’s study (first published online on 29 January 2020) (*16*) of six transmission pairs in Wuhan was higher than most of the later estimates. That estimate was applied as an assumed serial interval in 10 studies published in March and April 2020, (*54*, *55*, *57*, *60*, *71*-*76*) despite not being used in Li et al.’s own calculation of the reproduction number. (*16*) These early studies have been used to inform national and regional responses to the COVID-19 pandemic, and they demonstrate the importance of and reliance on early estimates to inform future research and public health decision-making.

Variations in the estimated reproduction number may also occur due to other assumptions applied in calculations. The initial estimate of the reproduction number of 0.3 assumed zoonotic transmission as the primary mode of transmission, based on the information available at the time. (*46*) The method applied may also influence the final estimate of the reproduction number. This is evident in the studies estimating the reproduction number of the Wuhan outbreak from December 2019 to mid-February 2020, which increased in later publications that used the same data sources and time periods. The reproduction number was estimated to be 2.2 in studies published in January and February 2020, (*16*, *48*) but increased to 4 in articles published in March and April 2020. (*62*, *77*, *78*)

The epidemiological parameters reviewed share some similarities to that of SARS and Middle East respiratory syndrome (MERS), two diseases caused by coronaviruses that have caused significant outbreaks in the early 21st century. The estimates of the range and mean of the incubation period of COVID-19 are similar to that of SARS (2–10 days, mean of 5–6 days) (*2*, *63*, *79*) and MERS (2–14 days, median of 5–6 days). (*79*, *80*) However, the estimated serial interval for COVID-19 is shorter than the observed intervals for SARS (8.4 days) (*63*) and MERS (7.6–12.6 days). (*80*, *81*) The later estimates of the COVID-19 serial interval published in April 2020 are shorter than the estimates for the incubation period, suggesting the potential for pre-symptomatic transmission, which has not been observed for SARS or MERS. (*63*, *80*, *82*) The estimated reproduction number of COVID-19 is similar to the estimates for the 2002–2003 SARS outbreak. (*63*)

This study has some important limitations. It provides a descriptive assessment and does not include meta-analysis or recalculations of results. The use of different methods and different outputs from each study limits the capacity for meta-analysis. This review may also be impaired by publication bias. Several included studies were based on small sample sizes, which led to imprecise results. The ongoing pandemic requires the active involvement of public health researchers to assess unfolding situations and advise on local responses. Fulfilling crucial roles as the pandemic unfolded may have limited the potential to publish findings, restricting our understanding of epidemic parameters in real time and reducing the representativeness of the results. This potential publication bias may also explain in part the overrepresentation of data from mainland China although COVID-19 has led to outbreaks worldwide. Nevertheless, the early published estimates included in this study have been used worldwide to inform public health responses, and they provide the best available evidence in the timeframe of this study.

Only studies written in English were included in this review. This excludes many early estimates written in Mandarin and Korean, which also limits the representativeness of this analysis. Furthermore, this analysis was limited to peer-reviewed published journal articles indexed in PubMed, which represents only a fraction of the literature published on the COVID-19 pandemic. The current pandemic has seen the proliferation of pre-print articles and increased attention on their results. Grey literature published by WHO, national governments and other organizations were also omitted. In times of emergency, pre-prints and grey literature may provide new information in a timely manner; however, this review focused only on estimations of epidemic parameters that have been subject to external peer review.

Pandemics are inherently uncertain times. The challenges of the ongoing COVID-19 pandemic are compounded by SARS-CoV-2 being a new pathogen, which public health and clinical professionals have had to rapidly assess, understand and respond to. Early estimates can provide useful interim guidance for public health decision-making. This is particularly true for transmission that is driven by biological characteristics, such as the incubation period. Epidemic characteristics that are influenced by human behaviours and public health interventions are less certain and require interpretation within the context of data collection and analysis of the study. Reliance on data from small sample sizes and specific settings is necessary in the context of an outbreak, but it also limits the generalizability of findings to other contexts.

Uncertainty in epidemic characteristics should not mean that we do not act. Although earlier estimates may rely on less-than-ideal sample sizes and sample structures, they are necessary to facilitate decision-making in a timely manner. However, reliance on the first estimates published may limit or bias our understanding of new data. The increasing availability of pre-print articles provides an outlet for urgent distribution of findings during an outbreak of a novel pathogen, provided preliminary findings are interpreted with caution before peer review. This study underscores the ongoing challenge and ever-present need for outbreak investigations and research to be both timely and frequently updated, to provide the best evidence to guide interventions. Further research is required to refine estimates of the serial interval and reproduction number, to improve our understanding of this pandemic in different contexts, and to provide reference values to enable a timely response to potential future outbreaks of COVID-19 and any future emerging coronaviruses and other potential pandemic diseases.
